# Indirect treatment comparison of odevixibat and maralixibat for the treatment of cholestatic pruritus in patients with Alagille syndrome

**DOI:** 10.57264/cer-2026-0138

**Published:** 2026-09-18

**Authors:** Emmanuelle Kaltenbach, Alice Guiraud-Diawara, Jérémie Schwarzbard, Cécile Artaud

**Affiliations:** 1 Ipsen, London, UK; 2 Ipsen, Paris, France

**Keywords:** Alagille syndrome, indirect treatment comparison, maralixibat, odevixibat, quality of life, tolerability

## Abstract

**Aim:**

Alagille syndrome (ALGS) is a rare genetic disorder associated with cholestasis and subsequent severe pruritus, which significantly impacts patient quality of life (QoL). Odevixibat (ODX) and maralixibat (MRX) are ileal bile acid transporter inhibitors approved for the treatment of cholestatic pruritus in ALGS. In the absence of head-to-head data, this study aimed to compare the efficacy, safety, and health-related QoL (HRQoL) associated with ODX and MRX.

**Materials & methods:**

A population-adjusted unanchored indirect treatment comparison was conducted using patient-level data from the Phase III ASSERT/ASSERT-EXT studies (ODX) and published data from the Phase IIb ICONIC study (MRX). A regression-adjusted matching approach was applied to adjust for cross-trial differences in baseline characteristics and estimate treatment effects. Outcomes assessed at week 48 included pruritus, serum bile acid (sBA) levels, HRQoL (PedsQL) and safety.

**Results:**

In adjusted analyses, ODX demonstrated numerically greater improvements in pruritus and reductions in sBA than MRX. ODX was associated with significantly greater improvements in HRQoL, including overall PedsQL scores (mean difference: 14.64; 95% CI: 5.70–23.58). ODX was better tolerated, with significantly lower rates of gastrointestinal adverse events (odds ratio [OR]: 0.22; 95% CI: 0.06–0.87) and abdominal pain (OR: 0.06; 95% CI: 0–0.83), and numerically lower rates of other adverse events.

**Conclusion:**

In this first indirect treatment comparison of ileal bile acid transporter inhibitors in ALGS, ODX demonstrated a differentiated clinical profile versus MRX, with significant benefits in HRQoL and tolerability, and consistent trends favoring better efficacy. These results may support informed treatment decision-making for patients.

## Plain language summary

**What is this article about?:**

Alagille syndrome (ALGS) is a rare genetic condition that affects the liver and other body organs. Many children with ALGS experience severe itching caused by a buildup in the blood of a substance called bile acid, which normally helps digest fats in the food we eat. Severe itching can be distressing, disrupt sleep and reduce quality of life. Two medicines, odevixibat and maralixibat, are approved to treat severe itching in ALGS, but there are no studies directly comparing them. This study compared the two medicines by using data from separate clinical trials. As the patients in each trial had different characteristics before treatment, researchers used statistical methods to adjust for these differences and make the comparison as fair as possible.

**What were the results?:**

The results showed that odevixibat led to greater, although not statistically significant, improvements in itching and reductions in bile acid levels than maralixibat. Children treated with odevixibat also had significantly greater improvements in health-related quality of life, including physical, emotional, and social well-being and sleep. In addition, odevixibat was better tolerated than maralixibat, with significantly fewer episodes of common side effects like diarrhea and abdominal pain. Better tolerability may lead to fewer pauses in treatment, help patients stay on treatment and reduce the impact on daily life and the burden on caregivers.

**Why is this important?:**

Overall, this first comparison of the two treatments for ALGS suggests that odevixibat may offer meaningful benefits for children with ALGS, which can help patients, caregivers and doctors make informed treatment decisions.

Alagille syndrome (ALGS) is a rare genetic, multisystemic disorder, with an estimated prevalence of 1 in 30,000 live births [1]. Genetic variants in *JAG1* and *NOTCH2* are reported in approximately 94.3% and 2.5% of patients, respectively [2]. These *JAG1* and *NOTCH2* variants cause disrupted Notch signaling, leading to hepatic manifestations, such as bile duct paucity and cholestasis, and extrahepatic manifestations, such as congenital heart disease and characteristic facial features [3]. Cholestasis often appears in infancy, causing elevated serum bile acid (sBA) levels and severe pruritus, the latter of which can be debilitating and is associated with sleep disturbance and reduced health-related quality of life (HRQoL) [3–5].

ALGS is a life-threatening disease; the mortality rate is approximately 12% and many patients with ALGS and cholestasis need a liver transplant before they reach adulthood, due to refractory pruritus, growth failure and progressive liver damage [3]. Indeed, liver transplantation is standard of care for patients with ALGS who have progressive cholestasis and decompensated portal hypertension; however, surgical intervention is highly invasive and associated with severe complications such as vascular thrombosis, severe bleeding, renal failure, neurologic complications, graft failure or sepsis [5–7].

Pharmacotherapies such as ursodeoxycholic acid and rifampicin have historically been used to relieve pruritus in patients with ALGS, but often with limited success [7]. The ileal bile acid transporter (IBAT) is located in the terminal ileum and mediates the return of ∼95% of bile acids to the liver, with the remaining excreted in feces [8,9]. Odevixibat (ODX) and maralixibat (MRX) are IBAT inhibitors that reduce reabsorption of bile acids to the liver; instead, they divert them to the colon for fecal excretion and, thus, reduce levels circulating in the blood [10–16].

ODX is approved in the US and EU for the treatment of cholestatic pruritus in patients with ALGS at least 12 or 6 months of age, respectively [10,11]. The efficacy and safety of ODX were confirmed in the Phase III, randomized, placebo-controlled ASSERT study (NCT04674761) and its 72-week extension (ASSERT-EXT; NCT05035030) [12,13]. MRX is approved in the US and EU for the treatment of cholestatic pruritus in patients with ALGS at least 3 or 2 months of age, respectively [14,15]. The efficacy and safety of MRX were confirmed in the Phase IIb placebo-controlled randomized drug withdrawal study ICONIC (NCT02160782) [16].

European Association for the Study of the Liver (EASL) guidelines recommend ODX or MRX in patients with ALGS and refractory cholestatic pruritus [7]. To date, there have been no direct head-to-head data assessing the efficacy, safety and HRQoL of ODX and MRX in ALGS or other cholestatic liver diseases. The objective of this indirect treatment comparison (ITC), therefore, was to compare the efficacy, safety and HRQoL outcomes in patients with ALGS treated with ODX or MRX, using clinically meaningful measures of treatment benefit. This may help inform appropriate treatment decision-making between patients, caregivers and healthcare professionals.

## Materials & methods

### Data sources

Clinical data for ODX and MRX were indirectly compared using a regression-adjusted matching approach, based on published methodology [17] and consistent with current ITC guideline recommendations, to adjust for prognostic factors known to affect outcomes (Figure 1). Data sources included patient-level data from the full analysis set of the Phase III ASSERT (study start: 19 March 2021/data cutoff: 29 September 2022) and ASSERT-EXT studies (study start: 3 September 2021/data cutoff: 7 February 2024) [12,13], and data from the overall study population of the Phase IIb ICONIC publication and supplement (study start: 28 October 2014/primary completion: 28 May 2020) [16,18]. ASSERT was a double-blind, randomized, placebo-controlled study with the primary end point assessing change from baseline in pruritus. ICONIC was an 18-week open-label study followed by a 4-week placebo-controlled, randomized drug withdrawal phase, and then by a 26-week stable dosing period; the primary end point assessed change in sBA level in patients who responded (≥50% sBA reduction) during the open-label period. The data available for pruritus scores, sBA levels, safety and Pediatric Quality of Life Inventory (PedsQL™) through the stable dosing phase in ICONIC publications made it possible to compare all of these outcomes with those in ASSERT and ASSERT-EXT. The comparison was performed at 48 weeks for the outcomes stated because it was the longest follow-up for both IBAT inhibitors.

**Figure 1.**
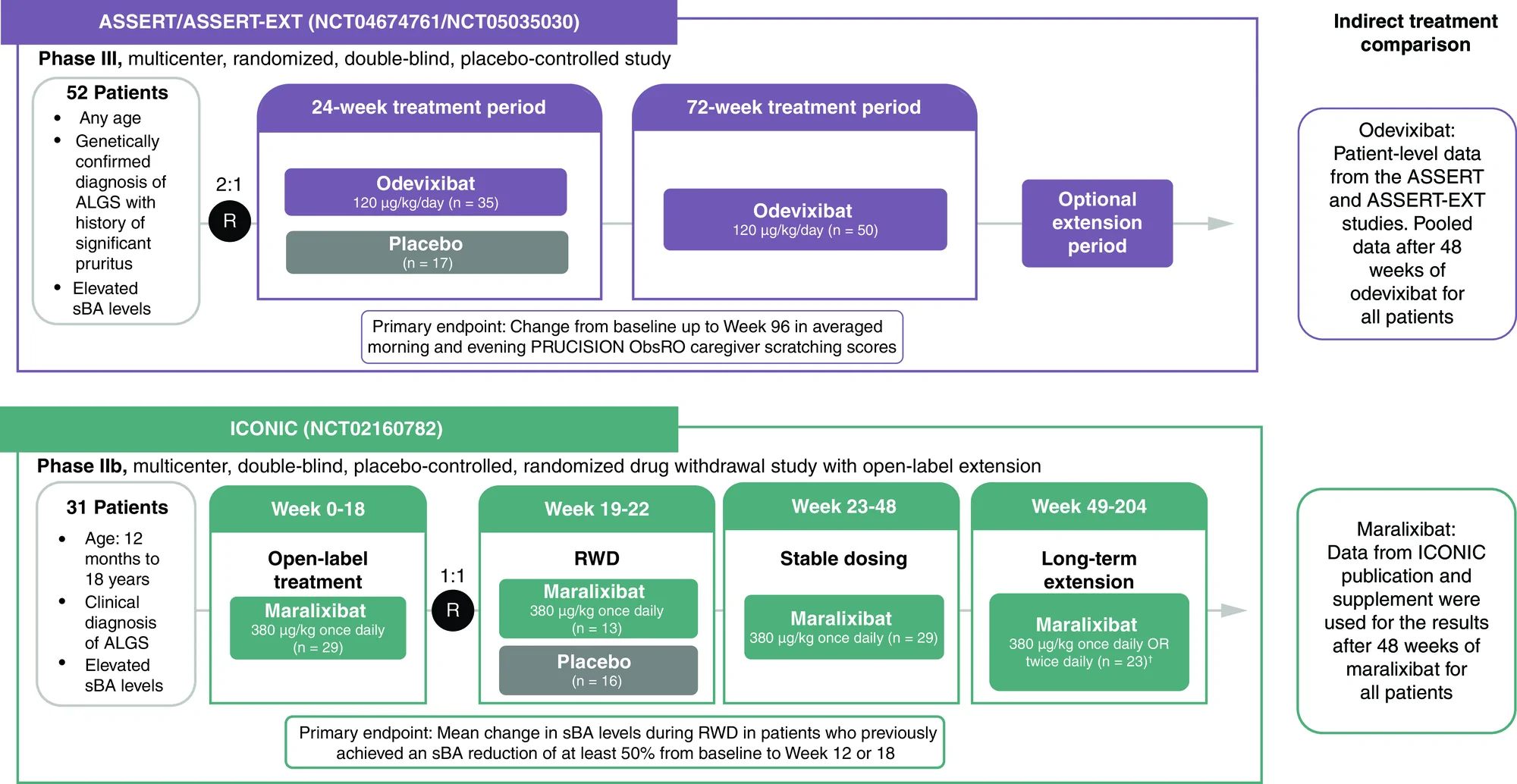
ASSERT, ASSERT-EXT and ICONIC study overview. ^†^Twice per day dosing started after Week 100. ALGS: Alagille syndrome; ObsRO: Observer-reported outcome; RWD: Randomized withdrawal; sBA: Serum bile acid.

### ITC outcomes

Clinically relevant efficacy and patient-reported outcome end points were assessed from baseline to week 48 (Table 1). The end points and time points for evaluation differed from the pre-specified primary end points of ICONIC and ASSERT and should be considered exploratory. Pruritus was measured using the observer-reported outcome in ASSERT and the itch-reported outcome in ICONIC. Despite apparent differences between the two assessment tools, they shared important similarities; both were observer-reported measures with a 5-point response scale ranging from 0–4 and assessed comparable aspects of pruritus (Supplementary Table 1). On this basis, they were considered sufficiently similar for an ITC, although this is acknowledged as a limitation. HRQoL was measured from baseline to week 48 by the parent-assessed PedsQL in both studies by total score and trouble sleeping score. Adverse events (AEs) that were incident during weeks 24 to 48 for ODX, and weeks 23 to 48 for MRX (the stable dosing period in ICONIC), were analyzed descriptively and reported as a proportion of patients experiencing the event.

**Table 1.** Clinically relevant study end points.

Outcome	End point
Pruritus	Change from baseline to week 48 in AM pruritus score Proportion of patients achieving ≥1-point reduction in AM pruritus score at 48 weeks Proportion of patients achieving a pruritus response (AM pruritus score ≤1) at 48 weeks
sBA	Change from baseline to week 48 in sBA level
PedsQL (parent)	Change from baseline to week 48 in total score Change from baseline to week 48 in trouble sleeping
AEs	Proportion of patients experiencing: ○ SAEs ○ GI AEs ○ Diarrhea ○ Vomiting ○ Abdominal pain

AE: Adverse event; AM: Morning; GI: Gastrointestinal; PedsQL: Pediatric quality of life inventory; sBA: Serum bile acid; SAE: Serious adverse event.

### Statistical analyses

A population-adjusted method was considered most appropriate based on published guidelines [19]. In situations where individual patient data are not available for the external comparator study, methods such as a matching-adjusted indirect comparison (MAIC), simulated treatment comparison or regression-adjusted matching approach can be applied.

A MAIC approach was first considered, including the following baseline characteristics for the adjustment of cross-trial differences, as they were described as potential prognostic variables and/or effect modifiers based on clinical input: age, sex, baseline sBA levels, baseline morning pruritus score and total bilirubin, with baseline PedsQL included for HRQoL analyses. However, the inclusion of total bilirubin and PedsQL as adjustment factors in the matching step resulted in a small effective sample size (ESS), precluding the use of a conventional MAIC approach for some outcomes. Alternatively, a regression-adjusted matching method was used, combining weighting and adjusted approaches to allow for further adjustment of remaining imbalances.

The lack of common placebo comparator precluded an anchored ITC. The validity of an unanchored ITC assumes that all relevant prognostic factors and treatment effect modifiers have been adequately adjusted for. The possibility of residual confounding due to unmeasured or unavailable factors cannot be excluded and is a potential limitation of the analysis.

The analysis was conducted in two steps. The first was the matching step, in which individual patients from the ASSERT/ASSERT-EXT trials were assigned weights such that the weighted mean baseline characteristics (age, sex, baseline pruritus score and baseline sBA level) matched those reported for the ICONIC trial population. Weights were estimated using a propensity score–type logistic regression model.

The second was the regression step. The individual weights obtained in the previous step and the following covariates were included in the regression model: age, sex, baseline pruritus score and baseline sBA levels, with baseline total bilirubin additionally included for sBA and safety outcomes, and baseline PedsQL total score included for HRQoL outcomes.

Weighted regression models were specified according to the outcome type. For binary outcomes, generalized linear models were used to estimate treatment effects as odds ratios (OR) with 95% CI and risk differences. OR were estimated using models with a binomial distribution and logit link, including treatment and baseline covariates, and by incorporating individual patient weights. Risk differences were estimated using models with a Gaussian distribution and identity link, with corresponding CI derived using robust (sandwich) variance estimators. For continuous outcomes, generalized linear models with a Gaussian distribution were used to estimate mean differences, incorporating the same covariates and weights.

For outcomes in which regression models did not converge (vomiting and abdominal pain), an MAIC approach was applied, using the same matching variables without regression adjustment. Matching and regression factors for the different outcomes are noted in the corresponding tables and figures.

No adjustments for multiplicity were performed and, in view of the multiple outcomes evaluated, findings should be interpreted as exploratory.

Additional details are provided in the Supplementary Appendix.

## Results

### Patients & baseline characteristics

The safety analysis included 45 patients in ASSERT and 31 patients in ICONIC who were participating at the start of the studies. The efficacy and HRQoL analyses included 45 patients in ASSERT and 27 patients in ICONIC who were participating at week 48 of the studies. Patient disposition in ASSERT/ASSERT-EXT and ICONIC is summarized in Supplementary Tables 2 & 3.

Before adjustment, patients receiving ODX were, on average, older and had lower pruritus scores, sBA levels and bilirubin levels at baseline. They also had higher PedsQL scores at baseline, indicating better HRQoL (Table 2). These cross-trial differences in age, sex, pruritus and sBA levels were addressed with the propensity score adjustment (Supplementary Tables 4–6). The remaining imbalance in total bilirubin levels and PedsQL scores was addressed in the regression model.

**Table 2.** Baseline characteristics for ASSERT and ICONIC analysis populations without propensity score adjustment per study outcomes.

Characteristic	All outcomes ASSERT ODX (n = 45) [12,13]	Efficacy and HRQoL outcomes ICONIC MRX (n = 27) [16,17]	Safety outcomes ICONIC MRX (n = 31) [16]
Mean age, years (SD)	6.01 (3.77)	5.70 (4.30)	5.4 (4.2)
Male (%)	23 (51.1)	18 (66.7)	19 (61.3)
Mean morning pruritus (SD)	2.81 (0.78)	2.90 (0.56)	2.9 (0.5)
Mean sBA, μmol/l (SD)	241.56 (119.11)	266.05 (213.86)	283 (211)
Mean total bilirubin, μmol/l (SD)	54.27 (47.63)	86.18 (80.88)	104.2 (98.9)
Mean PedsQL total score (SD)	69.95 (15.28)^†^	59.4 (17.0)	61.2 (17.3)

† Baseline data are available for 36 patients.

HRQoL: Health-related quality of life; PedsQL: Pediatric Quality of Life Inventory; sBA: Serum bile acid; SD: Standard deviation.

The ESS and proportion of patients with very small weights (≤0.2) for each analysis was as follows: pruritus analysis (ESS: 32.13%–0.0%), sBA analyses (ESS: 37.41%–0.0%), safety analyses (ESS: 37.50%–0.0%) and HRQoL (ESS: 23.47%–3.2%).

### Efficacy: Change in morning pruritus score

The mean change in morning (AM) pruritus score from baseline to week 48 for 39 patients receiving ODX in ASSERT/ASSERT-EXT was -2.04 (95% CI: -2.32 to -1.76; unadjusted population). The mean change in AM pruritus score from baseline to week 48 for 27 patients receiving MRX in ICONIC was -1.6 (95% CI: -2.1 to -1.1) [16]. After adjusting the ASSERT population for age, sex, baseline pruritus score and baseline sBA measurement, ODX demonstrated consistently greater improvements in AM pruritus score at week 48 with a mean difference of -0.50 (95% CI: -1.04–0.05) versus MRX (Table 3). Without population adjustment, 87.18% (34/39) of patients receiving ODX in ASSERT/ASSERT-EXT achieved a ≥1-point decrease in AM pruritus score at week 48 versus 75% (21/28) of patients receiving MRX in ICONIC [16]; after adjusting the ASSERT population for age, sex, baseline pruritus score and baseline sBA measurement, there was a numeric superiority for ODX (adjusted OR: 5.72; 95% CI: 0.91–36.12]; Figure 2). Without population adjustment, 66.7% (26/39) of patients receiving ODX in ASSERT/ASSERT-EXT achieved an AM pruritus score of ≤1 point at week 48 versus 42.9% (12/28) of patients receiving MRX in ICONIC [16]; after adjustment, there was a numeric superiority for ODX (adjusted OR: 2.35; 95% CI: 0.77–7.17). Overall, the adjusted pruritus results were consistent with the unadjusted results.

**Table 3.** Indirect comparison of odevixibat versus maralixibat for change from baseline to week 48 in pruritus score and serum bile acid levels.

Change from baseline to week 48	Population adjustment applied	Mean difference ODX vs MRX (n = 27) (95% CI)
Morning pruritus score (ESS = 32.13)	None	-0.44 (-1.01, 0.13)
	Regression-adjusted matching^†^	-0.50 (-1.04, 0.05)
sBA, μmol/l (ESS = 37.41)	None	-29.20 (-101.17, 42.76)
	Regression-adjusted matching^‡^	-26.94 (-97.04, 43.17)

† Propensity scores and regression based on age, sex, baseline pruritus score and baseline sBA.

‡ Propensity scores based on age, sex, baseline pruritus score and baseline sBA levels. Regression used age, sex, baseline pruritus score, baseline sBA and baseline total bilirubin levels.

ESS: Effective sample size; MRX: Maralixibat; ODX: Odevixibat; sBA: Serum bile acid.

**Figure 2.**
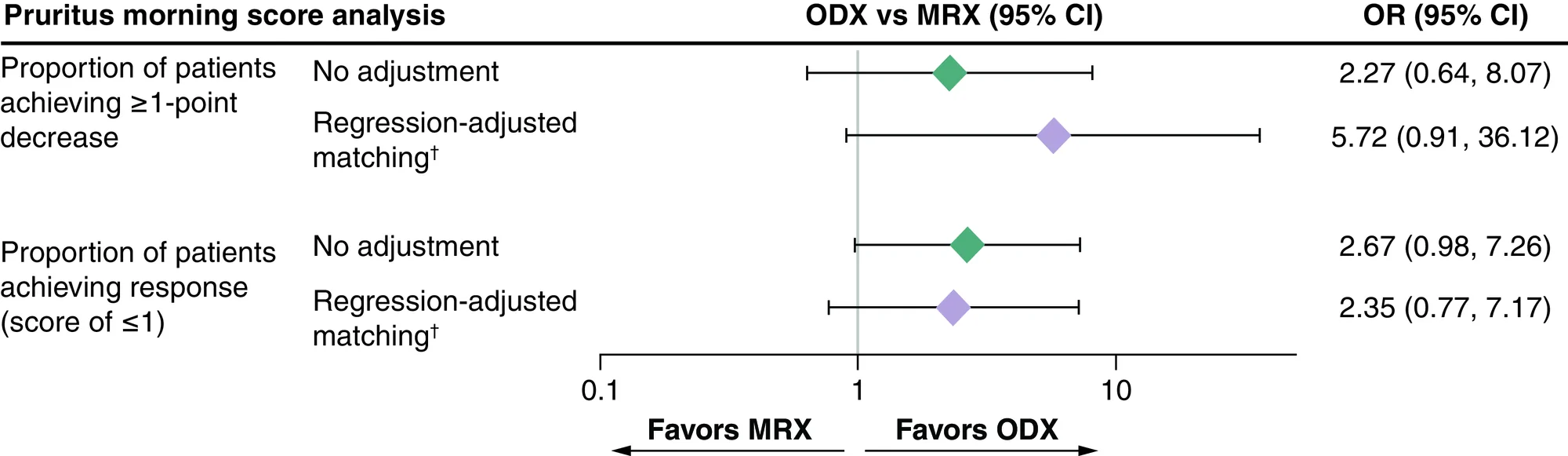
Indirect comparison of odevixibat versus maralixibat for the proportion of patients with improvements in pruritus scores at week 48 (effective sample size = 32.13). ^†^Propensity scores based on age, sex, baseline pruritus score and baseline sBA levels. Regression used the same factors. ESS: Effective sample size; MRX: Maralixibat; ODX: Odevixibat; sBA: Serum bile acid.

### Efficacy: change in sBA levels

Without adjustment, the mean change in sBA levels from baseline to week 48 for 44 patients receiving ODX in ASSERT/ASSERT-EXT was -125.20 μmol/l (95% CI: -155.02 to -95.39). The mean change in sBA levels from baseline to week 48 in 27 patients treated with MRX in ICONIC was -96 μmol/l (95% CI: -161.5 to -30.5) [16]. After adjusting the ASSERT population for age, sex, baseline pruritus score, baseline sBA measurement and baseline total bilirubin levels, patients receiving ODX had an additional reduction in sBA of 26.94 μmol/l versus patients treated with MRX. However, this difference was not statistically significant (95% CI: -97.04–43.17; Table 3). Overall, the adjusted results for sBA reduction were consistent with the unadjusted results.

### HRQoL outcomes

After 48 weeks of treatment in ASSERT/ASSERT-EXT, patients receiving ODX had improved HRQoL, with an unadjusted increase in PedsQL of 13.16 (95% CI: 6.31–20.01). After 48 weeks of treatment in ICONIC, patients receiving MRX had an increase in PedsQL total score of 8.9 (95% CI: 1.85–15.95) [18]. After adjusting the ASSERT population for age, sex, pruritus score, sBA measurement and PedsQL total score at baseline, ODX was associated with significantly superior improvement in patient HRQoL versus MRX at week 48, which was clinically meaningful and observed across overall HRQoL and sleep-related domains.

The adjusted mean difference in treatment effect on the PedsQL total score was 14.64 (95% CI: 5.70–23.58; Table 4). ODX was associated with numerically greater improvements in sleep in both the adjusted and unadjusted analyses (PedsQL trouble sleeping; Table 4). Overall, the adjusted results for HRQoL were consistent with the unadjusted results; however, only the former were significant.

**Table 4.** Indirect comparison of odevixibat versus maralixibat for regression-adjusted matching analysis of health-related quality of life from baseline to week 48 (effective sample size = 23.47).

Change from baseline to week 48	Population adjustment applied	Mean difference ODX (95% CI)
PedsQL total score	None	4.26 (-5.57, 14.10)
	Regression-adjusted matching^†^	14.64 (5.70, 23.58)
PedsQL trouble sleeping score	None	6.84 (-11.67, 25.35)
	Regression-adjusted matching^†^	8.39 (-12.91, 29.68)

† Propensity scores based on age, sex, baseline pruritus score and baseline sBA. Regression used age, sex, baseline pruritus score, baseline sBA levels and baseline PedsQL total score.

Statistically significant results based on a CI greater than 0 are in bold.

HRQoL: Health-related quality of life; MRX: Maralixibat; ODX: Odevixibat; PedsQL: Pediatric Quality of Life Inventory; sBA: Serum bile acid.

### Safety & tolerability outcomes

In ASSERT, 22% (10/45) of patients experienced a gastrointestinal (GI) AE during weeks 24 to 48 of ODX treatment. In ICONIC, 48% (14/29) of patients with available data experienced a GI AE during weeks 23 to 48 of the stable dosing period for MRX treatment [16]. After adjusting the ASSERT population for age, sex, pruritus score, sBA level and baseline total bilirubin levels, ODX was associated with significantly lower proportions of GI AEs (OR 0.22; 95% CI: 0.06–0.87), with a -27.84% (95% CI: -53.55 to -2.13) reduced risk of experiencing GI AEs versus MRX in ICONIC (Figure 3) [16].

**Figure 3.**
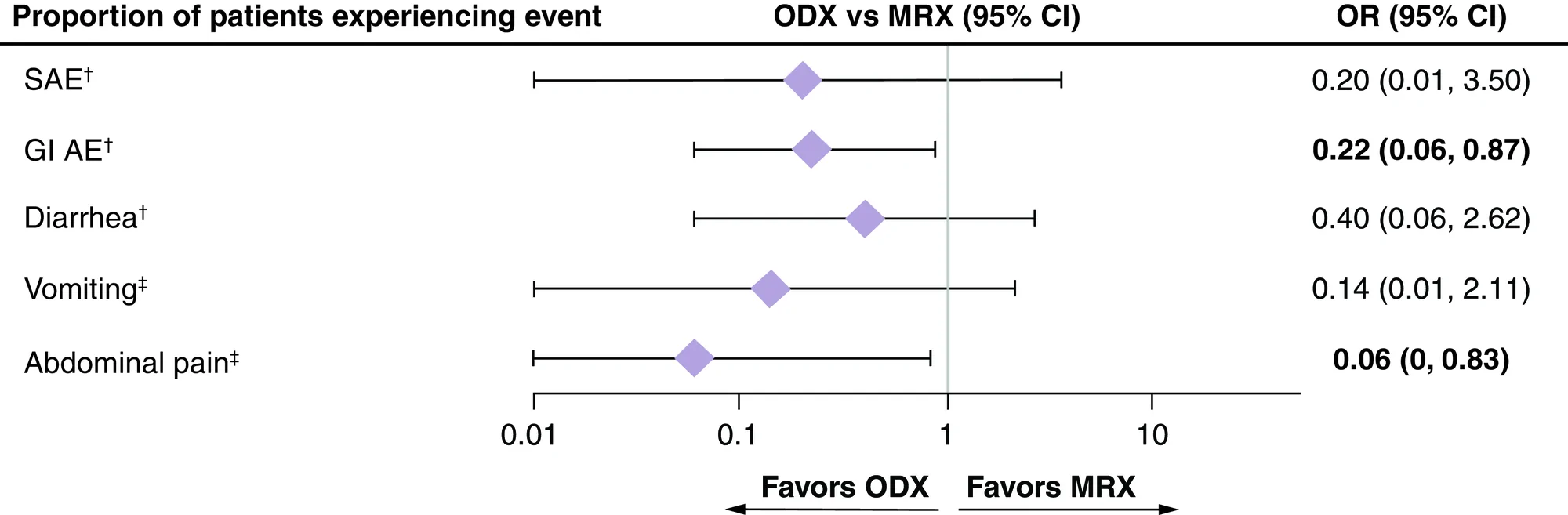
Indirect comparison of odevixibat versus maralixibat for adjusted analysis of adverse events with odevixibat and maralixibat (effective sample size = 37.50). Results in bold denote a statistically significant treatment effect. Unadjusted results were consistent with the regression-adjusted results (Table 5). ^†^Regression-adjusted matching population adjustment with propensity scores based on age, sex, baseline pruritus score and baseline sBA levels. Regression used age, sex, baseline pruritus score, baseline sBA and baseline total bilirubin. ^‡^MAIC population adjustment with propensity scores based on age, sex, baseline pruritus score and baseline sBA levels. AE: Adverse event; ESS: Effective sample size; GI: Gastrointestinal; MAIC: Matching-adjusted indirect comparison; MRX: Maralixibat; ODX: Odevixibat; SAE: Serious adverse event; sBA: Serum bile acid.

In ASSERT, 2% (1/45) of patients experienced abdominal pain during weeks 24–48 of ODX treatment. In ICONIC, 21% (6/29) of patients experienced abdominal pain during weeks 23–48 of the stable dosing period for MRX treatment [16]. After adjusting the ASSERT population for age, sex, baseline pruritus score, and baseline sBA level, ODX was associated with significantly lower proportions of patients experiencing abdominal pain (OR 0.06; 95% CI: 0–0.83), with a -19.13% (95% CI: -34.35 to -3.91) reduced risk (Table 5).

**Table 5.** Indirect comparison of odevixibat versus maralixibat for regression-adjusted matching analysis of adverse events with odevixibat versus maralixibat (effective sample size = 37.50).

Patients with an event during Weeks 23/24 and 48	Population adjustment applied	Odds ratio ODX vs MRX (95% CI)	Risk difference ODX vs MRX % (95% CI)
SAE	None	0.47 (0.11, 1.91)	-8.35 (-24.42, 7.71)
	Regression-adjusted matching^†^	0.20 (0.01, 3.50)	-1.98 (-23.49, 19.53)
GI AE	None	**0.31 (0.11, 0.84)**	**-26.05 (-47.92, -4.18)**
	Regression-adjusted matching^†^	**0.22 (0.06, 0.87)**	**-27.84 (-53.55, -2.13)**
Diarrhea	None	0.74 (0.20, 2.69)	-3.91 (-20.87, 13.05)
	Regression-adjusted matching^†^	0.40 (0.06, 2.62)	-3.65 (-24.19, 16.89)
Vomiting	None	0.20 (0.02, 1.99)	-8.12 (-20.01, 3.77)
	MAIC^‡^	0.14 (0.01, 2.11)	-8.70 (-20.44, 3.04)
Abdominal pain	None	**0.09 (0.01, 0.77)**	**-18.47 (-33.83, -3.11)**
	MAIC^‡^	**0.06 (0.00, 0.83)**	**-19.13 (-34.35, -3.91)**

† Propensity scores based on age, sex, baseline pruritus score, and baseline sBA. Regression used age, sex, baseline pruritus score, baseline sBA levels, and baseline total bilirubin levels.

‡ Propensity scores based on age, sex, baseline pruritus score, and baseline sBA levels.

Results in bold indicate a statistically significant treatment effect.

AE: adverse event; GI: gastrointestinal; MAIC: matching adjusted indirect comparison; MRX: maralixibat; ODX: odevixibat; SAE: serious adverse event; sBA: serum bile acid.

Without adjustment, a trend of improved tolerability was observed with ODX versus MRX, with numerically lower rates of serious AEs (SAEs), diarrhea and vomiting (Figure 3). Overall, the adjusted results for safety were consistent with the unadjusted results.

## Discussion

The ASSERT/ASSERT-EXT and ICONIC trials provide supportive evidence of benefit for the use of IBAT inhibitors in patients with ALGS, which has ultimately led to the approval of ODX and MRX for cholestatic pruritus [11–16]. There are no head-to-head data or robust ITC analyses comparing efficacy, safety, and HRQoL outcomes in patients with ALGS treated with ODX versus MRX. Therefore, this is the first analysis applying regression-adjusted matching; an established, published and recommended methodology in current ITC guidelines [19,20], which may help to inform clinical decision-making between ODX and MRX in patients with ALGS.

Population-adjusted ITC methods are used when trials evaluating different treatments include clinically heterogeneous populations, rendering naive comparisons biased and unreliable [19,20]. By adjusting for differences in key effect-modifying baseline characteristics, these methods aim to account for cross-trial heterogeneity and improve the validity of comparative effectiveness estimates. In doing so, they help to ensure that any observed differences in outcomes are more likely attributable to the treatments themselves rather than to underlying differences in patient populations. This strengthens the credibility of evidence for informed clinical decision-making. As it was not possible to adjust for all clinically relevant prognostic factors and effect modifiers using a conventional MAIC approach, an established method, also referred to as a regression-adjusted matching approach, was employed. This approach helps to mitigate residual bias arising from incomplete adjustment by combining two complementary adjustment strategies: weighting and outcome modeling regression techniques. Applying this method enabled adjustment of the ASSERT/ASSERT-EXT population to better align with the ICONIC population, with respect to key prognostic factors and effect modifiers, thus supporting the overall validity of this ODX and MRX comparison and its use to inform treatment decision-making in ALGS.

Overall, ODX demonstrated consistently greater improvements in AM pruritus scores, pruritus response rates and sBA reductions at week 48 versus MRX in patients with ALGS. However, results were not statistically significant and were associated with wide CIs, indicating uncertainty in a treatment effect difference between MRX and ODX. The analysis of PedsQL total score demonstrated that ODX also significantly enhanced patient HRQoL versus MRX, suggesting that clinical improvements with ODX are associated with better HRQoL from the patient perspective. Finally, safety results showed that ODX was overall better tolerated than MRX, with patients experiencing significantly lower rates of GI AEs and abdominal pain; there was also a trend toward fewer SAEs and less vomiting and diarrhea. Taken together, the present data build on the robust, pooled long-term ASSERT/ASSERT-EXT data, which demonstrate that ODX was associated with rapid and significant improvements in pruritus, significant reductions in sBA levels, and a well-tolerated safety profile from baseline up to 96 weeks of treatment in patients with ALGS [21].

GI AEs are the most common side effect with the IBAT inhibitor class, likely due to the increased delivery of bile acids to the colon following inhibition of the apical sodium-dependent bile acid transporter [12,16]. In this ITC analysis, there were significantly lower rates of GI AEs and abdominal pain with ODX versus MRX, and a trend for fewer SAEs with ODX. In ASSERT, no patients had an AE leading to discontinuation whereas [12] in ICONIC, one patient (3%) had AEs leading to discontinuation during the stable dosing period [16]. These findings are consistent with a lower incidence of GI AEs in patients treated with ODX than those treated with MRX, which is clinically relevant, particularly to treatment persistence, overall tolerability and daily disease burden. The GI AEs observed with ODX were generally manageable and rarely led to treatment discontinuation. Thus, despite the limited data on dose-target engagement relationships, ODX (120 μg/kg) appears to achieve greater efficacy than MRX (380 μg/kg), with improved pruritus relief and reductions in sBA levels, yet fewer GI side effects. One mechanistic hypothesis for the observed differences in GI tolerability involves potential differences in bile acid metabolism and microbiome effects; however, these mechanisms were not investigated in the present analysis. In the colon, bacterial conversion of primary to secondary bile acids strongly stimulates TGR5 receptors, thereby promoting intestinal motility, fluid secretion, and faster GI transit [22,23]. MRX has been associated with increased secondary bile acids, whereas these remain largely undetectable with ODX [24,25]. The resulting lower TGR5 stimulation may contribute to the more favorable GI tolerability observed with ODX. This, in turn, may be driven by the distinct physicochemical properties of the compounds, with ODX being a reversible acid with pH dependency and MRX being a permanent cationic base [10,14]; however, these differences were not investigated during this analysis and further research is needed to confirm this potential mechanism.

In this ITC analysis, it was demonstrated that patients had significantly improved HRQoL with ODX versus MRX (per PedsQL total score, which assesses physical, emotional, social and school functioning domains). This significantly improved HRQoL is likely due to improvements in pruritus severity, reduced sleep disturbance and a lower GI AE burden. Pruritus is often cited as the most debilitating symptom of ALGS [3,4] and together with GI tolerability, appears to play a key role in shaping patients' daily functioning and overall HRQoL. In line with this, EASL guidelines recommend the use of IBAT inhibitors (ODX or MRX) for the treatment of cholestatic pruritus in patients with ALGS [7]. Taken together, these ITC data provide supportive evidence of the comparative efficacy and safety of the two IBAT inhibitors available for the treatment of ALGS, and provide guidance for clinicians to make informed treatment decisions together with their patients.

Despite the methodologic advantages of population-adjusted ITCs in reducing bias, several inherent limitations warrant careful consideration when interpreting these findings. First, the differences in study designs of ASSERT/ASSERT-EXT and ICONIC are important to note. ASSERT was the only Phase III, randomized, double-blind, placebo-controlled trial in ALGS, while ICONIC was a placebo-controlled, randomized drug withdrawal period, Phase IIb study [11,16,21], meaning that the anchoring to placebo for MRX was not possible. The lack of anchor requires strong assumptions, including that absolute outcomes can be predicted from observed covariates, and that all relevant prognostic factors and effect modifiers are accounted for. Second, the absence of individual patient data from ICONIC introduces the potential for residual confounding due to unmeasured variables. Third, only two studies were included in the analysis, limiting the ability to reliably assess heterogeneity and, by extension, the consistency between sources of evidence (i.e., agreement across studies), while increasing the likelihood that results are driven by a single study. In addition, the indirect method used for comparison of pruritus scores is acknowledged as a limitation. Finally, both studies had small sample sizes; although this is expected in rare diseases, it further reduces the statistical power to detect meaningful differences. To mitigate these limitations in this analysis, all key prognostic factors available at baseline (including age, sex, pruritus, sBA levels and bilirubin levels) were included to minimize imbalance between studies. While this approach cannot replace a head-to-head randomized trial and some residual uncertainty may remain, ITCs are a well-established and widely accepted method to generate comparative evidence when no direct trials are available. When appropriately adjusted and cautiously interpreted, we believe this approach remains supportive of the comparison between the two studies, despite its acknowledged limitations.

A further consideration is whether observed differences in this ITC translate into clinically meaningful benefits. The difference in improvement in HRQoL with ODX versus MRX exceeded established thresholds for clinical relevance in pediatric populations (minimal clinically important difference of ∼4.5 points on the PedsQL total score). This may translate into observable improvements in day-to-day functioning across physical, emotional and social domains and reduced sleep disruption and caregiver burden. In real-world care, these aspects often drive treatment satisfaction, treatment persistence, and shared decision-making, particularly when efficacy end points (pruritus and sBA) appear directionally similar within statistical uncertainty. Further research, including real-world analyses of patients with ALGS who switch between IBAT inhibitors, may help clarify the clinical relevance of these findings in routine practice.

Enrolling participants in studies of a rare disease is challenging; real-world comparative studies may be more feasible than head-to-head trials and, to date, no head-to-head clinical data have evaluated different IBAT inhibitors in patients with ALGS or other cholestatic liver diseases. This is the first analysis to apply a regression-adjusted matching methodology to indirectly compare efficacy, safety and HRQoL outcomes of ODX versus MRX in patients with ALGS. ODX demonstrated a differentiated clinical profile versus MRX, characterized by significant improvements in GI tolerability and patient HRQoL, alongside consistent trends favoring improved efficacy. Although exploratory and hypothesis-generating, this unanchored population-adjusted ITC provides comparative insights between two available IBAT inhibitors in ALGS, and may support informed clinical discussion in the absence of head-to-head evidence.

## Summary points

- Alagille syndrome (ALGS) is a rare, life-threatening genetic disorder that causes cholestasis and severe itching (pruritus), which can disrupt sleep, daily activities and quality of life for children and their families.
- Odevixibat (ODX) and maralixibat (MRX) are both approved ileal bile acid transporter (IBAT) inhibitors for treating cholestatic pruritus in ALGS, but no head-to-head clinical trials have directly compared their impact on efficacy, safety or health-related quality of life.
- This study was a population-adjusted indirect treatment comparison of ODX and MRX, applying regression-adjusted matching to account for differences between patients enrolled in the separate ODX (ASSERT/ASSERT-EXT) and MRX (ICONIC) clinical trials.
- After adjustment, ODX showed numerically greater improvements in itch severity and reductions in serum bile acid levels than MRX, although these differences were not statistically significant.
- ODX was associated with significantly greater improvements in health-related quality of life, including overall Pediatric Quality of Life Inventory scores and domains related to physical, emotional, social and school functioning.
- Children receiving ODX also experienced significantly greater improvements in sleep, an important outcome given the impact of pruritus on nighttime symptoms.
- ODX demonstrated significantly better gastrointestinal (GI) tolerability than MRX, with lower rates of diarrhea and abdominal pain, and numerically fewer GI adverse events overall.
- Better tolerability may help children remain on treatment with fewer interruptions, while reducing the impact of treatment on daily life and the overall burden on caregivers.
- The differences in GI tolerability may be related to differences in how the two medicines affect bile acid metabolism and the gut microbiome; however, this was not investigated during this analysis.
- Overall, this population-adjusted unanchored indirect treatment comparison of ODX and MRX suggests that ODX offers a differentiated clinical profile, providing the first supportive comparative evidence of two available IBAT inhibitors in ALGS to potentially inform treatment decision-making between clinicians and their patients/caregivers.

## Supplementary Material


