# Supplementary material for: Indirect treatment comparison of odevixibat and maralixibat for the treatment of cholestatic pruritus in patients with Alagille syndrome
Source: J Comp Eff Res. 2026 Sep 18;15(10):e260138. doi: 10.57264/cer-2026-0138 (PMC13648047; doi:10.57264/cer-2026-0138)
Supplement: Supplementary file 1 [file cer-15-260138-s1.docx]

**Supplementary materials**

1. Additional statistical methods
2. Tables

**Additional statistical methods**

The first step in conducting regression-adjusted matching analysis involved deriving balanced weights such that the average baseline characteristics of ASSERT/ASSERT‑EXT after re-weighting match the published aggregate characteristics of ICONIC. A propensity score–type logistic regression equation was used to estimate weights; this equation predicted whether a given type of patient originates from ASSERT/ASSERT-EXT or ICONIC as a function of baseline characteristics. Specifically, weights were estimated by the odds calculated as, where is the vector of baseline variables included for matching. The *β* coefficients were determined by the method of moments rather than maximum likelihood (as is usually the case) because only the aggregated data for the x’s were available for the comparator populations. Individual patient weights were used to calculate the estimated sample size (ESS) after weighting patients as. The ESS is the number of independent non-weighted patients who would be required to give an estimate with the same precision as the weighted sample estimate. A small ESS indicates an irregular distribution of weights across patients, meaning that only a small fraction of patients drives the treatment effect.

Patient characteristics at baseline were summarized for the respective populations from ASSERT/ASSERT-EXT and ICONIC. Separate summaries by trial were displayed for patients before and after matching (the latter included patient weights). The ESS (which assesses the impact of re-weighting on the available statistical information in the individual patient data [IPD]), computed as the square of the summed weights divided by the sum of the squared weights, is displayed for ASSERT after matching. Re-weighted individual patient data from ASSERT were used for the analyses. Pseudo‑IPD on efficacy and safety binary endpoints were created from the external study (ICONIC). Estimates of the relative treatment effect comparing the index (ODX) versus the comparator (MRX) are presented in tables, including mean differences, odds ratios (ORs), or risk differences with 95% confidence intervals (CIs) and p-values.

All statistical models used ICONIC as the reference group. An OR >1 indicates a higher likelihood of the event occurring with ODX versus MRX (i.e., in favor of ODX), while an OR <1 indicates a lower likelihood of the event occurring with ODX (i.e., in favor of MRX). A 95% CI that does not include one or a p-value <0.05 indicates statistical significance. The opposite is true for outcomes where more events indicate worse outcomes, such as for safety.

**Tables**

**Supplementary Table 1.** PRUCISION vs ItchRO instruments. Both instruments include morning and bedtime pruritus assessments scored on a 0–4 scale and are administered via electronic diaries, with sleep disturbance also evaluated. In both tools, a ≥1-point reduction in observer-reported pruritus scores is considered a clinically meaningful change

| Trial | Instrument | Description | Questions asked | Ratings |
| --- | --- | --- | --- | --- |
| ASSERT / ASSERT-EXT | PRUCISION observer-reported outcome (ObsRO) | The ObsRO scratching, itching, and tiredness items use a 5-point pictorial response scale ranging from 0 to 4; higher scores indicate worse symptoms. | AM: How bad was your child’s worst scratching since he/she went to bed last night? PM: How bad was your child’s worst scratching since he/she woke up this morning? | With facial expression: 0 - No scratching 1 - A little scratching 2 - Medium scratching 3 - A lot of scratching 4 - Worst possible scratching |
| ICONIC | Itch-Reported Outcome Observer (ItchRO [Obs]) | Intended to measure cholestatic pruritus in children and includes a 5-point response scale that ranges from 0 to 4. Daily ItchRO scores are documented in a diary. | AM: How itchy was your child from when he/she went to bed last night until he/she woke up? PM: How itchy was your child all day today from the time he/she woke up until the time he/she went to bed? | Without facial expression: 0 - Not itchy at all 1 - A little bit itchy 2 - Somewhat/moderately itchy 3 - Very itchy 4 - Extremely itchy |

AM, morning; ItchRO, Itch Reported Outcome; ObsRO, Observer-Reported Outcome; PM, evening.

**Supplementary Table 2.** ICONIC patient disposition.

| ICONIC^1^ | n |
| --- | --- |
| Enrolled in open-label MRX treatment period | 31 |
| Discontinued due to AEs | 2 |
| Entered randomized drug withdrawal period | 29 |
| Allocated to receive MRX | 13 |
| Allocated to receive placebo | 16 |
| Received MRX after the randomized drug withdrawal period | 29 |
| Reasons for treatment discontinuation |  |
| AE | 1 |
| Consent withdrawn | 5 |
| Entered long-term extension | 23 |

^1^Gonzales E, *et al.* *Lancet* 2021;398:1581–92.
AE, adverse event; MRX, maralixibat.

**Supplementary Table 3.** ASSERT/ASSERT-EXT patient disposition.

| ASSERT^1^ | Placebo | ODX | Overall |
| --- | --- | --- | --- |
| Patients screened | - | - | 76 |
| Excluded | - | - | 24* |
| Assigned to randomized treatment | 17 | 35 | 52 |
| Completed randomized treatment | 17 | 35 |  |
|  |  |  |  |
| ASSERT-EXT | **Placebo/ODX** | **ODX/ODX** |  |
| Allocated to extension treatment | 17 | 33 | 50 |
| Reasons for treatment discontinuation |  |  |  |
| AE | 1 |  |  |
| Consent withdrawn | 1 | 2 |  |
| Liver transplant | 1 |  |  |
| Medication changed | 1 |  |  |
| Completed extension treatment | 13 | 31 | 44 |

^1^Ovchinsky N, *et al.* *Lancet Gastroenterol Hepatol* 2024;9:P632–45. Available from: https://doi.org/10.1016/S2468-1253(24)00074-8.
*14 participants did not meet SBA or pruritus inclusion criteria; 5 had ALT or total bilirubin concentrations higher than exclusion criteria thresholds; 3 did not meet other inclusion or exclusion criteria; 2 withdrew consent.
AE, adverse event; ALT, alanine aminotransferase; ODX, odevixibat; SBA, serum bile acid.

**Supplementary Table 4.** Baseline characteristics for pruritus and safety analyses at Week 48 (ASSERT full analysis set)

|  |  | Efficacy analyses | | Safety analyses | |
| --- | --- | --- | --- | --- | --- |
| Treatment | ASSERT 48 weeks, ODX (N=45) | ASSERT 48 weeks, ODX after matching (ESS=32.13) | ICONIC MRX (N=27) | ASSERT 48 weeks, ODX after matching (ESS=37.50) | ICONIC MRX (N=31) |
| Mean age, years (SD) | 6.01 (3.77) | 5.70 (3.55) | 5.70 (4.30) | 5.4 (3.3) | 5.4 (4.2) |
| Number of males (%) | 23 (51.1) | 23.5 (66.7) | 18 (66.7) | 25 (61.3) | 19 (61.3) |
| Mean morning pruritus (SD) | 2.81 (0.78) | 2.90 (0.66) | 2.90 (0.56) | 2.9 (0.7) | 2.9 (0.5) |
| Mean sBA, µmol/L (SD) | 241.56 (119.11) | 266.05 (117.02) | 266.05 (213.86) | 283.0 (126.6) | 283 (211) |
| Mean total bilirubin, µmol/L (SD) | 54.27 (47.63) | 55.87 (45.32) | 86.18 (80.88) | 59.2 (45.0) | 104.2 (98.9) |
| Mean PedsQL total score (SD) | 69.95 (15.28) | 71.25 (15.34) | 59.4 (17.0) | 72.6 (14.4) | 61.2 (17.3) |

ESS, effective sample size; MRX, maralixibat; ODX, odevixibat; PedsQL, Pediatric Quality of Life Inventory; sBA, serum bile acids.

**Supplementary Table 5**. Baseline characteristics for sBA analyses at Week 48 (ASSERT full analysis set)

|  |  | Efficacy analyses | |
| --- | --- | --- | --- |
| Treatment | ASSERT 48 weeks, odevixibat (N=45) | ASSERT 48 weeks, odevixibat after matching (ESS=37.41) | ICONIC maralixibat (N=27) |
| Mean age, years (SD) | 6.01 (3.77) | 5.70 (3.46) | 5.70 (4.30) |
| Number of males (%) | 23 (51.1) | 27.0 (66.67) | 18 (66.7) |
| Mean morning pruritus (SD) | 2.81 (0.78) | 2.90 (0.71) | 2.90 (0.56) |
| Mean sBA, µmol/L (SD) | 241.56 (119.11) | 266.05 (125.12) | 266.05 (213.86) |
| Mean total bilirubin, µmol/L (SD) | 54.27 (47.63) | 56.24 (43.14) | 86.18 (80.88) |
| Mean PedsQL total score (SD) | 69.95 (15.28) | 71.56 (14.00) | 59.4 (17.0) |

ESS, effective sample size; PedsQL, Pediatric Quality of Life Inventory; sBA, serum bile acids; SD, standard deviation.

**Supplementary Table 6**. Baseline Characteristics for HRQoL analyses at Week 48 (ASSERT full analysis set)

|  |  | HRQoL analyses | |
| --- | --- | --- | --- |
| Treatment | ASSERT 48 weeks, odevixibat (N=45) | ASSERT 48 weeks, odevixibat after matching (ESS=23.47) | ICONIC maralixibat (N=27) |
| Mean age, years (SD) | 6.01 (3.77) | 5.70 (2.57) | 5.70 (4.30) |
| Number of males (%) | 23 (51.1) | 17.7 (66.67) | 18 (66.7) |
| Mean morning pruritus (SD) | 2.81 (0.78) | 2.90 (0.69) | 2.90 (0.56) |
| Mean sBA, µmol/L (SD) | 241.56 (119.11) | 266.05 (123.22) | 266.05 (213.86) |
| Mean total bilirubin, µmol/L (SD) | 54.27 (47.63) | 60.13 (42.71) | 86.18 (80.88) |
| Mean PedsQL total score (SD) | 69.95 (15.28) | 70.96 (13.59) | 59.4 (17.0) |

ESS, effective sample size; PedsQL, Pediatric Quality of Life Inventory; HRQoL, quality of life; sBA, serum bile acids; SD, standard deviation.
